# Arabic Web-Based Information on Oral Lichen Planus: Content Analysis

**DOI:** 10.2196/49198

**Published:** 2024-03-19

**Authors:** Azzam AlMeshrafi, Arwa F AlHamad, Hamoud AlKuraidees, Lubna A AlNasser

**Affiliations:** 1 Department of Periodontics King Saud Medical City Riyadh Saudi Arabia; 2 Dental Services Ministry of National Gaurd Health Affairs Riyadh Saudi Arabia; 3 King Saud bin Abdulaziz University for Health Sciences Ministry of National Gaurd Health Affairs Riyadh Saudi Arabia; 4 King Abdullah International Medical Research Center Ministry of National Gaurd Health Affairs Riyadh Saudi Arabia; 5 Department of Dental Medicine Maple Clinic Dammam Saudi Arabia; 6 Department of Population Health King Abdullah International Medical Research Center Ministry of National Gaurd Health Affairs Riyadh Saudi Arabia

**Keywords:** oral lichen planus, health information, Arabic, medical information, information seeking, quality, online information, Arab, oral, inflammatory, inflammation, chronic, mouth, mucous membrane, mucous membranes, reliable, reliability, credible, credibility, periodontology, dental, dentist, dentistry

## Abstract

**Background:**

The use of web-based health information (WBHI) is on the rise, serving as a valuable tool for educating the public about health concerns and enhancing treatment adherence. Consequently, evaluating the availability and quality of context-specific WBHI is crucial to tackle disparities in health literacy and advance population health outcomes.

**Objective:**

This study aims to explore and assess the quality of the WBHI available and accessible to the public on oral lichen planus (OLP) in Arabic.

**Methods:**

The Arabic translation of the term OLP and its derivatives were searched in three general search platforms, and each platform’s first few hundred results were reviewed for inclusion. We excluded content related to cutaneous LP, content not readily accessible to the public (eg, requiring subscription fees or directed to health care providers), and content not created by health care providers or organizations (ie, community forums, blogs, and social media). We assessed the quality of the Arabic WBHI with three standardized and validated tools: DISCERN, *Journal of the American Medical Association* (*JAMA*) benchmarks, and Health On the Net (HON).

**Results:**

Of the 911 resources of WBHI reviewed for eligibility, 49 were included in this study. Most WBHI resources were provided by commercial affiliations (n=28, 57.1%), with the remainder from academic or not-for-profit affiliations. WBHI were often presented with visual aids (ie, images; n=33, 67.4%). DISCERN scores were highest for WBHI resources that explicitly stated their aim, while the lowest scores were for providing the effect of OLP (or OLP treatment) on the quality of life. One-quarter of the resources (n=11, 22.4%) met all 4 *JAMA* benchmarks, indicating the high quality of the WBHI, while the remainder of the WBHI failed to meet one or more of the *JAMA* benchmarks. HON scores showed that one-third of WBHI sources had scores above 75%, indicating higher reliability and credibility of the WBHI source, while one-fifth of the sources scored below 50%. Only 1 in 7 WBHI resources scored simultaneously high on all three quality instruments. Generally, WBHI from academic affiliations had higher quality scores than content provided by commercial affiliations.

**Conclusions:**

There are considerable variations in the quality of WBHI on OLP in Arabic. Most WBHI resources were deemed to be of moderate quality at best. Providers of WBHI could benefit from increasing collaboration between commercial and academic institutions in creating WBHI and integrating guidance from international quality assessment tools to improve the quality and, hopefully, the utility of these valuable WBHI resources.

## Introduction

The internet has revolutionized the visibility and accessibility of data [1]. Over half of today’s world population used the internet in 2021 [2]. Searching the internet for web-based health information (WBHI) was the third most frequent internet activity, and 6.75 million searches are performed daily for health information [3,4]. Often, WBHI is considered a “first aid” resource for health information and is used to compare diagnoses or treatment options [5,6], or to supplement insufficient time with a health care provider [6,7]. More seriously, some patients believe that WBHI is trustworthy and might defer or replace medical consultation or treatment from health care providers [6,8,9]. Lastly, recent evidence suggests that seeking WBHI could be associated with changes in health behavior or patient outcomes [6,8,9]. Therefore, ensuring the availability, accessibility, and quality of WBHI is essential to the well-being of individuals and the community.

The format and quality of WBHI vary substantially. The format ranges from health blogs/forums based on personal experiences offering unregulated information to peer-reviewed journal articles that provide complex data addressed to medical professionals [10,11]. Therefore, the quality of WBHI could vary considerably between the resources of WBHI. Some global research indicates that the quality of some WBHI targeting the public could be of low quality [10,11]. Evidence that examined Arabic WBHI often found that a considerable proportion of the WBHI had low quality, including but not limited to not disclosing the authorship of the information, outdated information, and lack of advice that WBHI should not replace a health care consultation [12-16]. Often, high-quality WBHI would require payment (ie, subscription) for access, exacerbating the inequalities in accessing WBHI for some individuals [10]. The evaluation of context-specific WBHI (ie, language) emerges as a public health priority that might address some health literacy inequality.

Oral lichen planus (OLP) is a prevalent chronic inflammatory mucocutaneous disease that frequently affects the oral mucosa. Between 0.5%-2% of the world’s population is affected by OLP [17-19]. In a review of studies conducted in Arab countries, OLP had a potential malignant transformation rate ranging from 0.4% to 6.5% [20]. Although there is no gold standard measure of the quality of WBHI, some international tools like the *Journal of the American Medical Association* (*JAMA*) benchmarks and Health On the Net (HON) are frequently used to examine the WBHI in different languages. Literature that evaluated the English content of WBHI regarding OLP using the aforementioned tools reported moderate accuracy and reliability [11]. A comprehensive evaluation of 122 Arabic health websites revealed that these websites varied substantially in meeting some industry benchmarks, like the HON code. For instance, 16% of the websites provided information on their advertising policies, while 73% provided justification for the content included within a website [21,22].

Though accessing high-quality WBHI can effectively increase the public’s knowledge, support health-related decision-making, and improve health-seeking behaviors or outcomes [8,23], no studies focused on evaluating OLP-related WBHI in Arabic. As such, the need arises to scrutinize the content and accuracy of Arabic WBHI related to OLP that is accessible to the public. This study assessed the availability and quality of OLP-related resources in Arabic.

## Methods

### Search Strategy

This study was a cross-sectional evaluation of Arabic WBHI on OLP. We searched for the Arabic translation of the keywords “oral lichen planus,” “treatment of oral lichen planus,” and their derivatives (علاج حزاز فموي, حزاز فموي منبسط,حزاز فموي مسطح ,حزاز فموي) in three main search engines, namely, Google, Yahoo, and Bing. We used the Boolean operator (OR) to link these terms but did not use any conditions of filters to mimic electronic research that patients or members of the public might perform on OLP.

We reviewed the first few hundred links on each platform for inclusion until links were no longer relevant to OLP. We included resources (ie, web pages) with information on OLP in Arabic. We excluded WBHI focusing on extra-oral lichen planus, scientific content requiring membership (eg, subscriptions) or directed to professionals (ie, specialty journals), community-based forums without professional guidance, social media posts, and results promoted or advertised by search engines. We also excluded resources that included video or audio content only with no accompanying text and results that were in .doc, .pdf, or .ppt format, as the public might be less likely to seek such resources. Two trained evaluators (AA and HA) simultaneously screened and evaluated the OLP Arabic WBHI resources and deferred to the senior author (AFA) in case of disagreement.

### Domains and Tools for Evaluating WBHI

The WBHI resources were assessed for content and quality. The content of the resources was categorized as reported previously by Ni Riordan and McCreary [24] and attached in Multimedia Appendix 1 [24]. Briefly, results were grouped according to the affiliation of the web page (commercial, nonprofit, governmental, or university/medical center), specialization (if a web page is either entirely or partially related to the searched topic), content type (medical facts, clinical trials, human interest stories, or question and answer), and content presentation (text, images/graphs, videos, and audio). The quality of the web pages was assessed with the DISCERN instrument [25], the *JAMA* benchmarks [26], and the HON code [27].

The DISCERN tool aims to empower consumers when making treatment choices by evaluating the quality of written health information (ie, publications). DISCERN is a validated 16-item instrument, with each item rated on a 5-point scale (1=did not fulfill item to 5=complete fulfillment of item) and is arranged in three sections. The first section (items 1-8) addresses the reliability of the publication, the second section (items 9-15) assesses the quality of the information related to treatment choices, and the third section consists of one item (item 16) that gives an overall quality rating of the content [25,28,29].

*JAMA* benchmarks assess the accountability of WBHI by examining the following domains of resources: authorship (providing authors and their affiliations), attribution (eg, citations) of the source of information provided, disclosure (revealing any ownership, sponsorship, or conflict of interest in providing the WBHI), and currency (ie, updates of the WBHI) [26].

The HON tool evaluates the reliability and credibility of health websites. HON asks reviewers to evaluate a given web page regarding eight sections: authority, complementarity, confidentiality, attribution, justifiability, transparency, financial disclosure, and advertising policy [30]. The reviewer will score each item from 0% (did not fulfill the specific item) to 100% (total fulfillment of that item), while the HON final score is an average of scores across the eight items.

### Statistical Analysis

Data were extracted, coded, and cleaned in Excel (Microsoft Corporation), then analyzed using SPSS Statistics (Version 24.0; IBM Corp). The data were summarized using frequency and percentage distribution for the categorical variables and means with SDs for the continuous variables. We classified WBHI by type of affiliation into commercial and academic (including nonprofit, medical center, and governmental), then compared the quality of WBHI across these groups using bivariate analyses. To facilitate comparison with previous literature, the HON score was categorized into an ordinal variable as such: scores <50% (low credibility), 51%-75% (moderate credibility), and scores >75% (high credibility). Fleiss κ was used to calculate the interrater reliability of the reviewers in assigning the HON score as a categorical variable [31]. Lastly, to synthesize information from all three quality measures, we created a composite variable as follows: high-quality WBHI means the resource satisfied at least 3 of the *JAMA* benchmarks, had a HON score ≥75, and had a DISCERN overall score ≥3; otherwise, the WBHI was considered low-quality.

### Ethical Considerations

This study was exempt from ethical review since it used publicly available data sources that do not include any patient-identifying data.

## Results

### Overview

**Figure 1:**
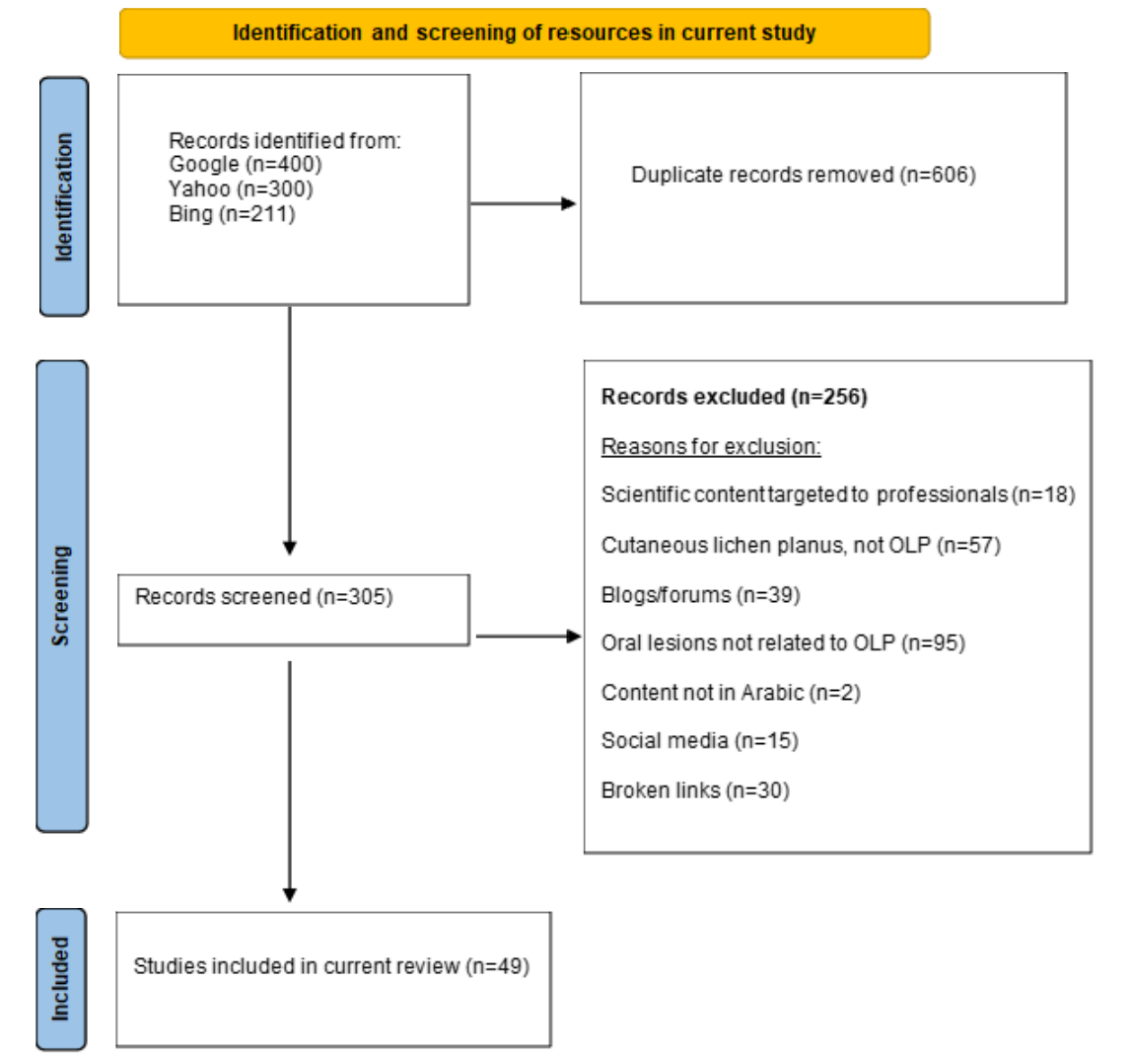
Flowchart of web-based health information resources' identification, screening, and inclusion (n=49). OLP: oral lichen planus.

The keywords returned the highest number of hits in Google (n=56,770), followed by Yahoo (n=5188) and Bing (n=1910). We reviewed the first 400 results on Google, 300 on Yahoo, and 211 on Bing until the web pages were unrelated to OLP. After removing duplicated results, a total of 49 results were included in this study. The most frequent reason for exclusion was results about oral lesions other than OLP (Figure 1). The interrater reliability measure indicated a high agreement between the evaluators in assigning HON scores (κ=0.826, 95% CI 0.823-0.828).

### Content Assessment

More than half of the web pages had commercial affiliations (n=28, 57.1%), that is, WBHI provided by for-profit clinics with clear advertisements for treatment or establishments (Table 1). Conversely, 43% (n=21) of the WBHI resources had academic affiliations (nonprofit, medical centers, and governmental affiliations). Only one-fifth of the web pages (n=11, 22%) were focused exclusively on OLP, rather than lichen planus in general. The vast majority of Arabic OLP WBHI was in the format of medical facts (n=42, 85.7%), and most WBHI included images of OLP (n=31, 63%; Table 1).

**Table 1:** Content assessment of Arabic web-based health information on oral lichen planus (OLP) using Ni Riordan and McCreary’s [24] method (n=49).

Categories		Web pages, n (%)
**Content affiliation**		
	Commercial	28 (57.1)
	Nonprofit	19 (38.8)
	Medical center	2 (4.1)
	Governmental	0 (0.0)
**Content specialization**		
	Entirely related to OLP	11 (22.3)
	Partially related to OLP	38 (77.6)
**Content format**		
	Medical facts	42 (85.7)
	Human stories	2 (4.1)
	Question and answer format	4 (8.2)
	Clinical trials	1 (2.0)
**Content presentation**		
	Included images	31 (63.3)
	Included videos	2 (4.1)
	Included audio	1 (2.0)
	Text only	15 (30.6)

### Quality Assessment by DISCERN

The overall mean score of DISCERN for the included WBHI was rather low (mean 2.61, SD 1); however, the average rating of single items varied substantially (Table 2). Items with the highest DISCERN scores described the publication’s aims and the alternative OLP treatment options. There was no evidence to indicate meaningful differences in the DISCERN scores between WBHI presented by commercial and academic affiliations.

**Table 2:** Overall and stratified average (SD) of DISCERN scores by affiliation type (n=49).

Domain and DISCERN item		All (N=49), mean (SD)	Academic (n=21), mean (SD)	Commercial (n=28), mean (SD)	*P* value
**Reliability**					
	Q1. Explicit aims	3.37 (1.0)	3.61 (0.7)	3.19 (1.1)	.07
	Q2. Attainment of aims	3.27 (1.0)	3.17 (1.1)	3.42 (0.7)	.27
	Q3. Relevance	3.18 (1.1)	3.47 (0.92)	2.96 (1.10)	.07
	Q4. Explicit sources	2.20 (1.8)	2.76 (1.94)	1.78 (1.57)	.06
	Q5. Explicit date	1.73 (1.4)	2.05 (1.5)	1.5 (1.17)	.13
	Q6. Balanced and unbiased	2.69 (1.5)	2.57 (1.50)	2.78 (1.47)	.66
	Q7. Additional sources	1.92 (1.2)	1.90 (1.22)	1.92 (1.27)	.96
	Q8. Areas of uncertainty	2.08 (1.3)	3.09 (1.13)	1.32 (0.77)	*<.001^a^*
**Treatment options**					
	Q9. How treatment works	2.57 (1.5)	2.47 (1.28)	2.64 (1.63)	.84
	Q10. Benefits of treatment	2.08 (1.3)	1.95 (0.86)	2.17 (1.56)	.85
	Q11. Risk of treatment	1.92 (1.2)	1.95 (0.86)	1.89 (1.47)	.24
	Q12. Effect of no treatment	1.67 (1.4)	1.42 (1.20)	1.85 (1.48)	.25
	Q13. Effect on quality of life	1.24 (0.7)	1.09 (0.43)	1.35 (0.78)	.18
	Q14. All alternatives described	3.37 (1.9)	3.38 (1.93)	3.35 (1.88)	.96
	Q15. Shared decision	3.20 (1.8)	2.61 (1.49)	3.64 (1.80)	*.04*
	Q16. Overall rating of the source	2.61 (1.0)	2.61 (1.16)	2.60 (0.91)	.98

^a^Italics indicate a statistically significant result.

### Quality Assessment by *JAMA* Benchmarks

Only 22.4% (n=11) of the WBHI resources met all 4 *JAMA* benchmarks, indicating high quality, and another 24.5% (n=12) met 3 of the 4 benchmarks. Overall, most WBHI fulfilled the “Disclosure” criteria, while less than half of the web pages fulfilled the “Attribution” criteria (Table 3). Academic affiliations satisfied the authorship and attribution more often than commercial affiliations but were less current than commercial resources (Table 3).

**Table 3:** Overall and stratified distribution of the fulfillment of *Journal of the American Medical Association (JAMA)* benchmarks and Health On the Net (HON) scores among reviewed web-based health information (WBHI; n=49).

			All (n=49), n (%)		Commercial affiliation (n=28), n (%)		Academic affiliation (n=21), n (%)		*P* value	
***JAMA* benchmarks fulfilled^a^**										
	Authorship	27 (55.1)		14 (50.0)		13 (61.9)		.56		
	Attribution	18 (36.7)		7 (25.0)		11 (52.4)		.07		
	Currency	33 (67.3)		20 (71.4)		13 (61.9)		.55		
	Disclosure	40 (81.6)		23 (82.1)		17 (80.9)		>.99		
	Fulfilling 3 benchmarks	12 (24.5)		6 (21.43)		6 (28.57)		.74		
	Fulfilling all 4 benchmarks	11 (22.4)		6 (21.43)		5 (23.81)		>.99		
**HON score (range 0-100)^b^**										.09
	<50	10 (20.4)		8 (28.6)		2 (10)				
	51-75	25 (51.1)		15 (53.6)		10 (46.6)				
	>75	14 (28.6)		5 (17.9)		9 (43.9)				
High-quality WBHI^c^			7 (14.29)		2 (7.14)		5 (23.81)		.12	

^a^*JAMA* comparisons denote raw percentages comparing the relative contribution by affiliation type within each *JAMA* benchmark.

^b^HON comparison denotes column contribution by affiliation type across levels of HON scores.

^c^High-quality WBHI denotes a WBHI resource that satisfied at least 3 of the *JAMA* benchmarks, had a HON score >75, and had an overall DISCERN score ≥3; otherwise, a resource was labeled as low-quality.

### Quality Assessment by HON

The average HON score was significantly higher for academic affiliation sources of WBHI than commercial affiliations (mean 71, SD 14 vs mean 56, SD 15; *P*<.001). When HON scores were examined as an ordinal variable, almost one-third (n=14, 29%) of the WBHI resources scored higher than 75, indicating high credibility of the WBHI, with more contributions from academic rather than commercial affiliations (Table 3).

### Overall Quality Assessment

When WBHI information was classified by collective score combining all three quality instruments, it was found that 1 in every 7 resources (n=7, 14.3%) had high quality, and most of this high-quality evidence came from academic affiliations (n=5, 23.8% vs n=2, 7.1% from commercial affiliations; Table 3). However, the difference in the distribution of quality levels across affiliation types was not significant (Fisher exact test *P*=.12).

## Discussion

### Principal Findings

This study evaluated the content and quality of the Arabic WBHI on OLP available to the public using prevalidated standardized tools. The results indicated that approximately one-third of the WBHI had high scores, corresponding to higher quality, as indicated by the *JAMA* and HON tools. The Arabic WBHI on OLP was often presented as a narration of medical facts and frequently included audiovisual aids to enhance the consumer’s comprehension of health information, and most were provided by commercial affiliations. To the best of our knowledge, this is the first study to evaluate Arabic WBHI on OLP. As OLP can be a chronic condition with occasional atypical forms and a potential for malignant transformation, patients are likely to supplement their clinical consultations with WBHI [7,17,32,33]. Hence, this evaluation of the quality of WBHI on OLP in Arabic is essential and timely.

The Arabic WBHI on OLP in this study included a higher percentage of resources deemed to be of higher quality compared to Arabic WBHI on other diseases, including periodontal diseases and breast and oral cancers [12,14,34]. For instance, in this study, 1 in every 5 WBHI sources had fulfilled all 4 *JAMA* benchmarks, while recent results by Halboub et al [16] and Al-Ak’hali et al [34] found the same for 8% and 4%, respectively, of the websites they reviewed. The association of higher quality with resources classified as academic was noted in previous studies evaluating Arabic WBHI [34]. Lastly, DISCERN scores in this study were slightly higher than those reported by Alakhali et al [14] on oral cancer. However, these studies evaluated different diseases, and the higher scores could reflect “better” WBHI on OLP compared to oral cancer or an inherent subjectivity in the DISCERN instrument by virtue of having a Likert scale.

Most of the WBHI resources we reviewed had more commercial affiliations than affiliations to medical or research centers. This generally agrees with the literature where commercial affiliations were the most prolific providers of WBHI [12,13,34]. This is consequential as the web pages with a commercial affiliation often include advertising of certain treatments or care providers. Some evidence suggests that advertising negatively affects the credibility of the information offered and individuals’ trust in the source of WBHI [35]. Moreover, compared to academic affiliations, we observed a lower quality among WBHI by commercial affiliations, as evidenced by the HON, DISCERN, and *JAMA* benchmarks scores. While these differences in quality scores did not always reach statistical significance (possibly due to the relatively small sample size), they indicate a consistent trend among commercial affiliations across all the quality tools used.

Of the WBHI we reviewed, the authority and attribution were the least fulfilled *JAMA* domains—that is, resources rarely mentioned the authors and the references of the WBHI. This was in line with previous studies on Arabic WBHI on oral cancer, periodontal diseases, and epilepsy [13,34]. This highlights an area of potential improvement as the authority of the author (ie, the level of expertise of the person writing the information) was found to increase the credibility and trust in the content of the WBHI [35]. Moreover, other evidence from an Arabic-speaking country showed that participants prioritized information provided by health care professionals over other sources [9]. Our findings, along with conclusions from other Arabic WBHI, are useful for developers of WBHI resources to promote transparency when creating web-based content to maximize the benefit of WBHI to the public.

Enhancing the content and quality of Arabic WBHI is pivotal in optimizing its effectiveness and influence on patients’ well-being. For example, research reported that 49% of the participants in a US-based national survey used WBHI as a first resource to address health concerns [36]. Another study from Saudi Arabia reported that the patients who sought diabetes mellitus–related WBHI, compared to nonseekers, were more health conscious and showed a positive trend of better self-care [8]. Three critical issues must be highlighted when we combine evidence from this study with conclusions from similar research on Arabic WBHI. First, the quality of available resources in Arabic is moderate at best, leaving the public at a disadvantage as most of the evidence is deemed low-quality. Second, developers and providers of WBHI of digital health content must invest in the quality of WBHI in Arabic to empower the public [37]. This could be accomplished by benchmarking the finalized content against the quality tools frequently used in the relevant literature to build more robust, impactful WBHI. Third, it is imperative to establish policies and guidelines that guarantee the quality of Arabic WBHI that are produced, disseminated, or promoted for the benefit of the public.

### Strengths and Limitations

This study reviewed the content of three major search engines, potentially covering the majority of OLP-related WBHI that the public could find, access, and use. Additionally, we used validated and standardized tools to evaluate the quality and content of OLP health information on the web, enhancing the comparability with research elsewhere. However, this study could not assess Arabic readability due to some challenges related to the available tools and our belief that readability in Arabic is context specific and best explored through research that uses community participatory methods (ie, focus groups). Future efforts should strive to include the readability of the Arabic WBHI from both the providers’ and the consumers’ perspectives. Lastly, the WBHI we evaluated included published web pages up to mid-2020, coinciding with the height of the COVID-19 pandemic. The COVID-19 pandemic increased the demand for digital resources in general, which might have positively affected the quality of WBHI in general in response to the increased demand for web-based information. However, given the comparison of our results to other recent evidence on Arabic WBHI post COVID-19 [14,34,38], we assume that the Arabic WBHI content did not experience a change of a magnitude large enough to substantially alter our conclusions regarding the quality of Arabic WBHI related to OLP available to the public.

### Conclusion

This study evaluated the quality of Arabic WBHI on OLP available to the public, using several validated and standardized tools, namely, *JAMA*, DISCERN, and HON. The results indicated that the quality of the WBHI is moderate at best, with only 1 in 7 resources scoring simultaneously high on all three resources. Commercial affiliations of the WBHI provided more content than academic affiliations; however, the content delivered by the former was of lower quality. Therefore, the collaboration between commercial and academic WBHI providers could improve the quality of the OLP resources offered to the public. Lastly, providers of WBHI in Arabic could benefit from integrating guidance from international quality tools to enhance the quality and, hopefully, the utility of these valuable WBHI resources.

## Appendix

Multimedia Appendix 1 Riordan and McCreary content categorization.

## Abbreviations

HON: Health On the Net
*JAMA*: *Journal of the American Medical Association*
OLP: oral lichen planus
WBHI: web-based health information

## Data Availability

Data are available from the corresponding author upon request.
