# Supplementary material for: Arabic Web-Based Information on Oral Lichen Planus: Content Analysis
Source: JMIR Form Res. 2024 Mar 19;8:e49198. doi: 10.2196/49198 (PMC10988371; doi:10.2196/49198)
Supplement: Multimedia Appendix 1 [file formative_v8i1e49198_app1.docx]

**Appendices**

**Appendix A**

Riordan and McCreary Content Categorization [24]:

| **Category** | **Types** |
| --- | --- |
| Affiliation of the webpage | Commercial |
|  | Nonprofit |
|  | Governmental |
|  | University/medical center |
| Specialization | Entirely related to the searched topic |
|  | Partially related to the searched topic |
| Content type | Medical facts |
|  | Clinical trials |
|  | Question and answer |
|  | Human interest stories |
| Content presentation | Text |
|  | Images/graphs |
|  | Videos |
|  | Audio |
